# Leveraging COVID-era innovation for cervical cancer screening: Clinician awareness and attitudes toward self-sampling and rapid testing for HPV detection

**DOI:** 10.1371/journal.pone.0282853

**Published:** 2023-03-09

**Authors:** Natalia M. Rodriguez, Luke P. Brennan, Layla Claure, Lara N. Balian, Victoria L. Champion, Michele R. Forman

**Affiliations:** 1 Department of Public Health, College of Health and Human Sciences, Purdue University, West Lafayette, Indiana, United States of America; 2 Weldon School of Biomedical Engineering, College of Engineering, Purdue University, West Lafayette, Indiana, United States of America; 3 Indiana University Simon Comprehensive Cancer Center, Cancer Prevention and Control Program, Indianapolis, Indiana, United States of America; 4 Formerly at Department of Nutrition Science, College of Health and Human Sciences, Purdue University, West Lafayette, Indiana, United States of America; University of Macerata: Universita degli Studi di Macerata, ITALY

## Abstract

Cervical cancer screening rates are declining in the US, with persistent disparities among vulnerable populations. Strategies to better reach under-screened communities are needed. The COVID pandemic sparked major shifts in healthcare delivery, including the accelerated development and adoption of rapid diagnostic testing, broadened access to remote care, and growing consumer demand for self-testing, which could be leveraged for cervical cancer. Rapid tests for the detection of Human Papillomavirus (HPV) have the potential to improve cervical cancer screening coverage, and if coupled with patient-collected cervicovaginal samples, create an opportunity for self-testing. The objectives of this study were: 1) to examine whether COVID influenced clinician perspectives of rapid testing as a screening modality; and 2) to assess clinician awareness, perceived benefits and limitations, and willingness to adopt point-of-care HPV testing, patient self-sampling, and rapid HPV self-testing with self-collected samples. The methodology adopted consisted of an online cross-sectional survey (n = 224) and in-depth interviews (n = 20) were conducted with clinicians who perform cervical cancer screening in Indiana, ranked in the top ten states for cervical cancer mortality and with marked disparities across socio-demographic groups. The main findings show that about half the clinicians reported that the COVID pandemic had influenced their views on rapid testing as a screening modality both positively (greater public acceptability of rapid testing and impact on patient care) and negatively (concerns regarding accuracy of rapid tests). The majority of clinicians (82%) were willing to adopt rapid HPV testing at the point-of-care, while only 48% were willing to adopt rapid HPV self-testing with self-collected samples. In-depth interviews revealed provider concerns around patients’ ability to collect their own sample, report results correctly, and return to the clinic for follow-up and other preventive care. Addressing clinician concerns about self-sampling and rapid HPV testing, such as ensuring that rapid tests include sample adequacy controls, is necessary to mitigate barriers to adoption for cervical cancer screening.

## Introduction

Cervical cancer screening remains one of the most effective methods in reducing incidence and mortality; yet recent estimates indicate that 23% of people eligible for cervical cancer screening in the US are not up-to-date with screening, a significant increase from 14% in 2005 [1]. The United States failed to meet the Healthy People 2020 cervical cancer screening target of 93%, which was then lowered to 83.4% for 2030 due to the expectation that rates would be difficult to improve [2]. Disparities in screening rates persist, particularly among racial and ethnic minorities, particularly Hispanic and Asian populations, sexual and gender minorities, rural populations, individuals with lower educational attainment or income, and those who are uninsured [1,3,4]. Potential drivers of these disparities cut across social, cultural, psychological, and institutional factors and include financial barriers, cultural beliefs about sexual health, mistrust in the healthcare system, lack of knowledge or awareness of cervical cancer, fear, embarrassment, or discomfort of screening, among others [1,3,4].

Historically, US Preventive Services Task Force cervical cancer screening guidelines recommended a Pap test, either alone every 3 years or combined with an HPV test every 5 years, for women aged 30–65 years. Since 2018, these guidelines now include primary HPV testing, which is performed on its own without a Pap test. Primary HPV testing detects high-risk HPV DNA, which is responsible for most cervical cancer cases, and is now the preferred testing method for women aged 25–65 among several organizations including the American Cancer Society and World Health Organization [5,6]. The 2022 President’s Cancer Panel Report on closing gaps in cancer screening [7] also includes recommendations emphasizing a transition to primary HPV testing, which would enable innovative testing modalities such as HPV self-sampling and rapid testing, that could facilitate screening for vulnerable and under-screened populations.

While the COVID pandemic disrupted preventive healthcare and contributed to a decline in cancer screening [8], it also precipitated the development, evaluation, and adoption of technological innovations like rapid diagnostic tests that could be leveraged to improve cervical cancer screening. Combined with other COVID-era healthcare advances like expanded telehealth, patient self-collected samples, and broadened access to home services, such innovations make HPV point-of-care (POC) testing and even self-testing possible through rapid HPV tests that are under development but not approved as yet [9–14].

The pandemic led to the rapid advancement of POC diagnostic technology and to growing consumer demand for self-tests with written instructions with explanatory figures for a patient’s self-collection. Self-tests for COVID were first approved by the FDA in November 2020, with use peaking at 11% in January 2022 from 2% in October 2021 [15]. For cervical cancer screening, HPV testing through self-collected cervicovaginal specimens, or self-sampling, could also potentially enable at-home self-testing. Several studies and meta-analyses have found that, compared with clinician-collected samples, self-collected HPV samples showed high diagnostic accuracy [16–18]. Highly acceptable or even preferred among women over clinician-collected samples [19–23], HPV self-sampling has garnered attention for its potential to increase screening by circumventing barriers to the Pap smear, and ease of use outside of a clinic.

While the self-sampling literature focuses mostly on mail-in and laboratory-based testing (i.e. not rapid testing), and while HPV rapid tests under development could be years from real-world application in the US context, understanding clinician perspectives on new cervical cancer screening modalities, such as HPV POC and self-testing, is essential to inform ongoing and future development of these technologies, and their eventual implementation in ways that address key barriers and reduce cervical cancer disparities. Indiana is among the top ten states with the highest rates of cervical cancer mortality, with marked disparities among Black, Hispanic, and rural populations. With HPV vaccination rates in Indiana below the national average [24], increasing screening coverage remains critical for cervical cancer prevention and addressing persistent disparities through the utilization of new technologies that could address barriers to screening for underserved populations. The objectives of this study are: 1) to examine whether COVID influenced clinician perspectives of rapid testing as a screening modality; and 2) to assess clinician awareness, perceived benefits and limitations, and willingness to adopt POC HPV testing, patient self-sampling, and rapid HPV self-testing with self-collected samples for cervical cancer screening.

## Methods

This convergent mixed-methods study was designed as an online cross-sectional survey and in-depth interviews with clinicians who conduct cervical cancer screening in Indiana. Quantitative survey items explored clinician awareness, perceived benefit, and willingness to adopt POC HPV testing, patient self-sampling, and patient self-testing with a rapid HPV test. Qualitative interviews provided insights on clinician’s specific perceived benefits and limitations of these innovative screening modalities, reasons why clinicians were willing or unwilling to adopt them, and whether the COVID pandemic influenced their overall perspective of rapid testing as a screening modality.

**Fig 1:**
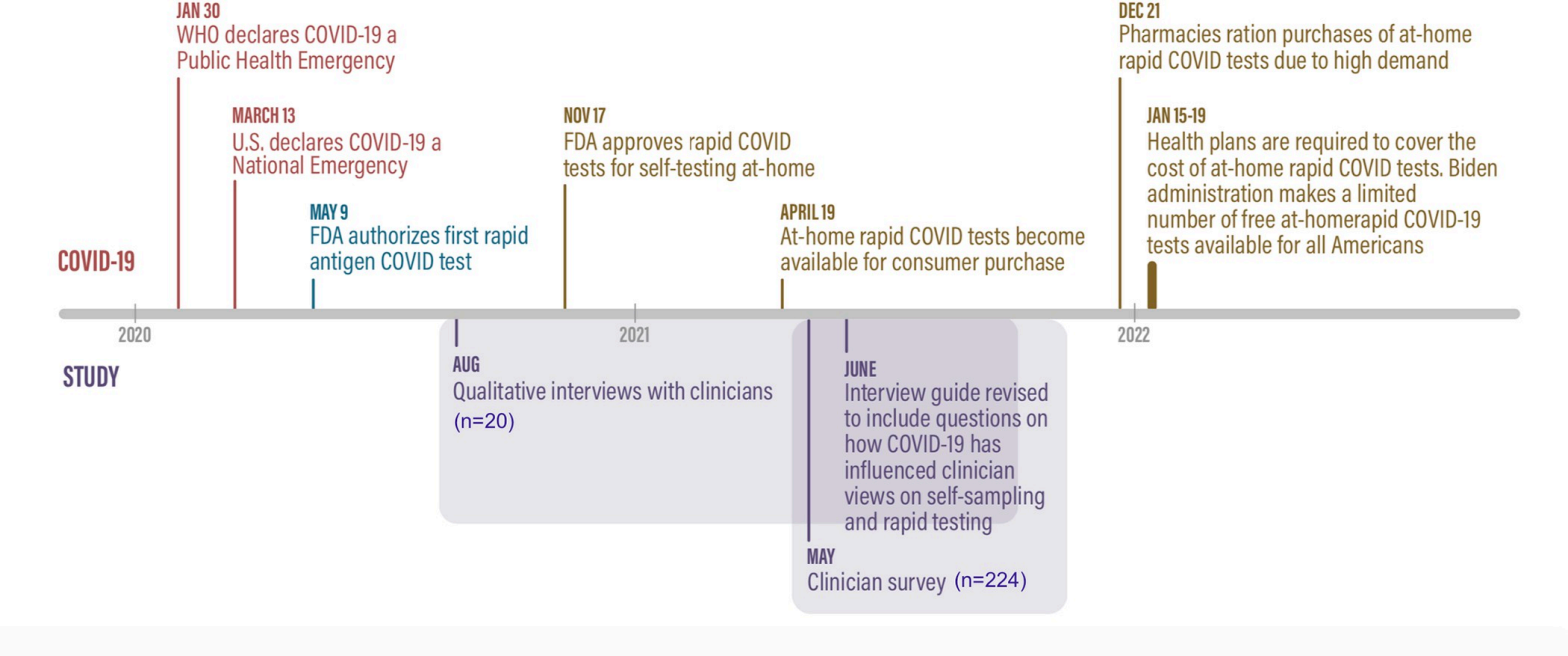
Timeline of study and COVID rapid testing developments.

As COVID self-testing became more widely adopted, the first few interviews informed the addition of survey items and interview questions specifically on whether and how COVID had influenced clinician views on self-sampling and self-testing in addition to POC testing. See Fig 1 for a study timeline along with COVID testing developments in the US.

### Survey questionnaire

Survey recruitment occurred in two waves, and the sample was restricted to Indiana clinicians who performed cervical cancer screening on at least one asymptomatic average-risk woman aged 21–65 years in the past month. The online survey was programmed into Qualtrics and advertised to the research team’s network of contacts that practice among underserved groups by posting in the Indiana Cancer Consortium and the ACOG district V newsletters, and emailing invitations through the Indiana University Simon Comprehensive Cancer Center’s Office of Community Outreach and Engagement, federally qualified health center (FQHC) partners, and Planned Parenthood clinicians in Indiana. This first wave began May 5^th^, 2021 and closed October 5^th^, 2021. In order to increase our sample size and collect responses from a broader sample of Indiana physicians and nurse practitioners in OB/GYN, family practice, and internal medicine specialties, a second wave was launched October 11, 2021 and closed November 17, 2021, where the same Qualtrics survey was distributed by Dynata, a market research firm. All survey respondents volunteered and were compensated upon completion of the survey with a $20 electronic gift card. Informed consent from participants was obtained electronically for the survey questionnaire and verbally for the in-depth interviews prior to participating in the research. All participant information was kept in password protected files only available to the study team. This study was approved by the Institutional Review Board at Purdue University (protocols: IRB-2019-132; IRB-2021-12; IRB-2021-617).

The self-reported questionnaire included items about demographic characteristics, professional information, POC testing, self-sampling, and self-testing for HPV detection, among other topics as part of a larger study about cervical cancer screening. A literature search was conducted for other surveys of clinician perspectives on cervical cancer screening, and adaptations of previously validated survey items [25–30] were used whenever possible. In the absence of validated items, the investigator-generated questions were developed with two iterations of survey drafts tested among a group of clinicians in the sampling frame population and pilot tested for clarity, clinical accuracy, and to ensure that the items were measuring the intended concept. The final survey instrument had 77 questions and took approximately 15 minutes to complete.

For each testing modality (POC, self-sampling, self-testing with a rapid test) respondents were primed with the clinical scenario of “a 35 year-old asymptomatic patient that had a normal last screening test (normal Pap/HPV-negative) 5 years ago, like all of her previous screening tests.” Respondents reported their familiarity with each testing modality, using a 5-point Likert scale from “This is my first time hearing about it” to “I currently use this frequently”. To gauge user-perceived benefit, respondents were asked the extent to which they believed each testing modality would improve or not improve cervical cancer screening, on a 4-point scale ranging from ‘Would not improve’ to ‘Would greatly improve’. Finally, to assess willingness to adopt each testing modality, respondents were asked to rate their agreement on a 5-point Likert scale from “strongly agree” to “strongly disagree” with the statement “I would support adopting [testing modality] in my practice as the preferred cervical cancer screening method for asymptomatic average-risk women ages 30–65.” Respondents that believed a method could improve screening but disagreed with supporting its adoption were prompted to answer a free-response item to explain why. All respondents were queried “Do you think COVID has influenced your view of rapid testing as a screening modality (in any way)” and those who selected ‘yes’ were prompted with the optional free-response item “How has COVID influenced your view of rapid testing.”

### Quantitative data analysis

Both waves were combined for the analysis. Of 310 total eligible respondents who initiated the survey, 271 completed it; however, 47 responses were from clinicians who practice outside of the state of Indiana and were excluded from the analysis, yielding 224 total responses. Descriptive statistics of frequencies and percentages, or means and standard deviations are reported as appropriate. Free-response data were reported as quotations, with the COVID free response data categorized manually into topical themes using Microsoft Excel.

### Qualitative interviews

Semi-structured in-depth interviews were conducted to glean additional qualitative insights on clinician experiences, perceived benefits and limitations, and reasons for their willingness or unwillingness to adopt POC HPV testing, patient self-sampling and self-testing with a rapid HPV test, among other topics as part of a larger study about cervical cancer screening. Recruitment for the interviews initially occurred through email invitations sent to our team’s network of clinicians in Indiana, and survey respondents who consented to being contacted for a follow-up interview were also invited to interview by the study team. We conducted virtual interviews via Zoom with 20 clinicians from August 2020 to October 2021. Interviews lasted 30 to 60 minutes and were audio recorded and transcribed verbatim using a digital transcribing platform, Otter.ai. Interview participants volunteered and were compensated upon completion of the interview with a $25 electronic gift card. Transcripts were reviewed and edited for accuracy by research assistants.

### Qualitative coding and analysis

The transcripts were thematically analyzed applying a combination of deductive and inductive coding by two independent coders using Nvivo software. The interview guide was used for deductive analysis of major themes and to create the initial codebook. Open coding was conducted whereby keywords and phrases in the codebook were assigned to interview sections, followed by axial coding, where patterns between and within interviews were mapped and inductive analysis was used to identify emerging subthemes [31]. The two coders met and discussed their coding to ensure for intercoder consistency and to reach consensus on coding. Any differences in coding or theme definitions were discussed among the full study team until consensus was reached.

## Results

### Clinician sample

A total of 224 eligible clinicians completed the online questionnaire. Respondent characteristics are reported in Table 1 and included physicians (n = 110, 49%), nurse practitioners (n = 107, 48%), medical assistants and physician assistants (n = 7, 3%), representing clinical specialties of family medicine (n = 150, 67%), obstetrics/gynecology (n = 36, 16%), and internal medicine (n = 26, 12%). Most respondents (n = 133, 62%) perform cervical cancer screening on at least 10 patients or more per month, and reported majority patient populations were 69% White, 12% Black, 6% Hispanic, and 13% other or unsure. The sample of survey respondents who participated in interviews (n = 20) included physicians (n = 11, 55%) and nurse practitioners (n = 9, 45%); in family medicine (n = 11, 55%), obstetrics/gynecology (n = 2, 10%), internal medicine (n = 2, 10%) and other (family planning, adolescent medicine, n = 5, 25%). Most interviewees (n = 13, 65%) were female and non-Hispanic white (n = 12, 60%).

**Table 1:** Survey respondent demographics (n = 224).

	N (%)
Gender	
Female	162 (72)
Male	57 (25)
Clinician race/ethnicity	
White, non-Hispanic	175 (78)
Black, non-Hispanic	7 (3)
Hispanic/Latinx	15 (7)
Asian	15 (7)
Other*	12 (5)
Clinician type	
Nurse Practitioner	107 (48)
Physician	110 (49)
Clinical specialty	
Obstetrician/Gynecologist	36 (16)
Family Medicine	150 (67
Internal Medicine	26 (12)
Years in practice (*Mean (SD))*	
	*14*.*6 (10*.*3)*
Practice type	
Private practice	25 (11)
Group Practice*	97 (43)
Hospital*	23 (10)
Community health clinic	67 (30)
Other	12 (5)
Patient load (patients per day)	
Less than 10	13 (6)
10–19	98 (44)
20–29	97 (43)
30 or more	16 (7)
Federally Qualified Health Center (FQHC)	
Yes	74 (33)
No	126 (56)
Unsure	24 (11)
Clinic geographic location	
Rural	47 (21)
Urban	71 (32)
Suburban	106 (47)
Majority patient payment method	
Private insurance/HMO	86 (38)
Medicaid	99 (44)
Uninsured/Self-pay	14 (6)
other/Unsure*	25 (12)
Majority patient race/ethnicity	
White, non-Hispanic	154 (69)
Black, non-Hispanic	27 (12)
Hispanic/Latinx	13 (6)
Other/Unsure*	30 (13)
Monthly cervical cancer screenings performed:	
Less than 10 patients	85 (38)
10 patients or more	139 (62)

*collapsed answer choices.

Gender: Excluded 5 ’prefer not to answer’ respondents.

Clinical Training: Excluded 3 ’physician assistant’ and 4 ’other’ respondents.

Clinical Specialty: Excluded 11 ’Other’ and 1 ’Gynecologic Oncologist’ respondents.

#### Influence of COVID on clinician views of rapid testing as a screening modality

More than half of respondents reported having their view of rapid testing as a screening modality influenced by COVID (n = 133, 52%). Of these, 87 responded to the optional free-response question ‘How has COVID influenced your view of rapid testing’ and their responses were coded into positive and negative influences and categorized across three themes: impact on patient care, accuracy, and acceptability of rapid testing. These themes aligned with those identified in in-depth interviews, and data below are both from survey free-responses and in-depth interviews.

### Impact on patient care

Clinicians felt that rapid testing impacted patient care in both positive and negative ways. Delivering quick results at the POC allowed clinicians to expedite and facilitate linkage to follow-up care. Rapid testing at-home through patient self-testing gave patients privacy and autonomy while conducting their tests and reduced screening barriers for those who were unwilling or unable to attend an actual office visit. Additionally, the option for telehealth visits made it easier to go over self-test results and decreased barriers to care. As one interviewee stated, “*Everybody has been given a taste of what things could actually be like*. *And the way that we can decrease barriers to care… so I think that COVID-19 has really showed us with the increase of telehealth and patients being able to do more things from home… that things like rapid testing… would definitely increase the rates of patients getting the care that they need*, *and the patient being comfortable accessing that care because they can do it on their own in their home and their own timeframe on their own phone etc*.*(Nurse Practitioner*, *Family Medicine*, *Female)”* However, clinicians also felt that self-testing also had the potential to negatively impact patient care as it also meant: “*Fewer patients want to come in face-to-face*” and may miss opportunities for other preventive care.

### Accuracy

The vast majority of concerns reported by clinicians regarding rapid testing as a screening modality focused on the accuracy of rapid tests based on their experiences during COVID. Clinicians felt that rapid tests tend to have lower sensitivity and can be faulty. Some reported that since they felt the tests were not as accurate, they had *“less faith in the results”* and became more skeptical about rapid screening tests in general. Some providers only trusted positive results from patient self-tests because collecting an inadequate sample was perceived as a common cause of false negative results, “*I don’t 100% trust the negative results*. *If I get a negative result on a patient that is pretty symptomatic*, *I still have to do a regular test*, *right*. *(Nurse Practitioner*, *Family Medicine*, *Female)”* Nevertheless, clinicians felt that rapid tests were a “*good way to get quick results*, *although a lot of times it may not be 100% accurate*, *a great way to screen a large population quickly*.”

### Acceptability of rapid testing

Clinicians reported that the increased availability and normalizing of rapid tests during the COVID pandemic increased their openness to using rapid testing as a screening tool, and many now see rapid POC testing as a favorable and acceptable tool which made getting results simple and quick. Clinicians also shared during interviews that self-testing at-home during the pandemic minimized exposure for both patients and medical personnel, “*I mean*, *at some point during this we were actually directing people how to give themselves their own depo shots*, *so that they didn’t have to come into the clinic and so… I think it’s really advanced like rapid testing at home and in the privacy of places …definitely we changed our model of healthcare based on minimizing exposure (Nurse Practitioner*, *Obstetrician/Gynecologist*, *Female)”* and it increased the acceptability of having less patient contact, *“I think I’m probably more accepting of having a test that maybe doesn’t require as much contact with the patient*, *or means they don’t have to come into the clinic*. *You know even if potentially …clinic screening is preferred if patients have this option… if they can’t come in*, *COVID has probably impacted my desire to have things like that*. *(Nurse Practitioner*, *Obstetrician/Gynecologist*, *Female)”* Not all interviewees felt as favorably, one participant expressing that *“because something is quicker does not make it better*.”

#### Clinician attitudes towards rapid HPV testing for cervical cancer screening

**Fig 2:**
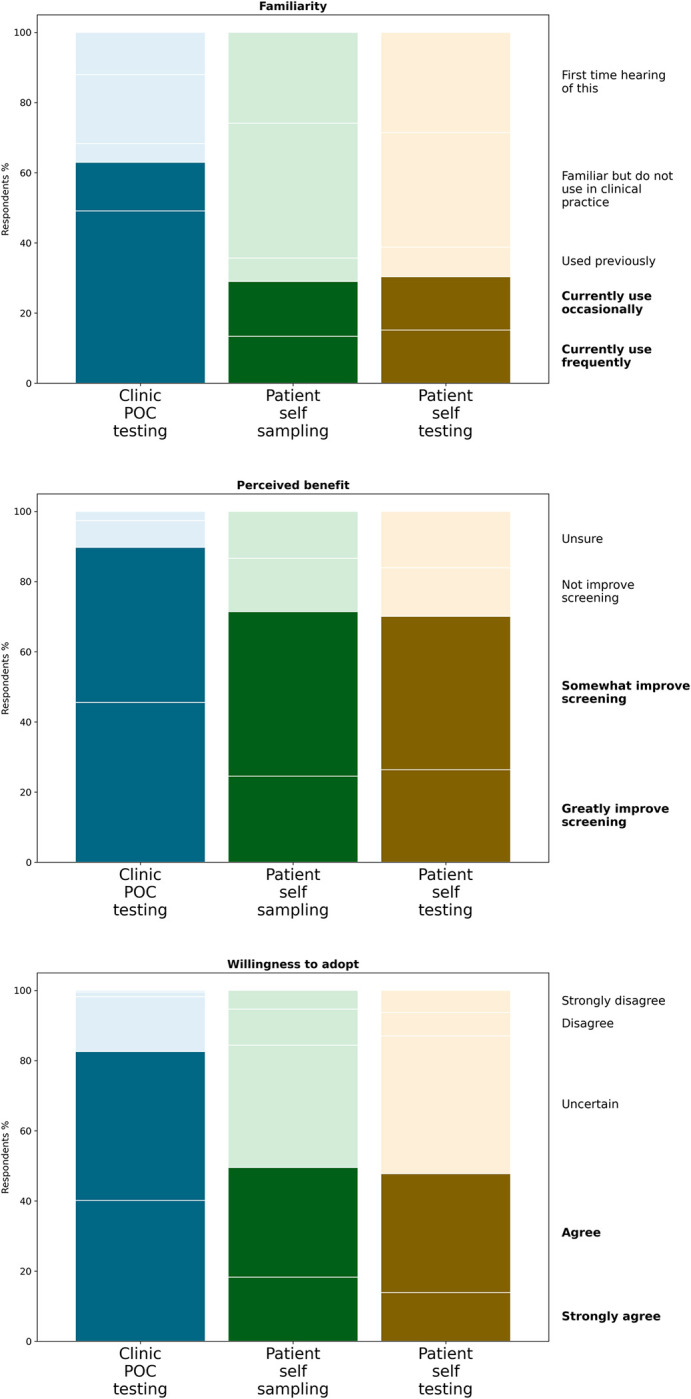
Clinician familiarity, perceived benefit, and willingness to support adoption of different cervical cancer screening modalities. For each testing modality (POC, self-sampling, self-testing) clinician familiarity, using a 5-point Likert scale from “This is my first time hearing about it” to “I currently use this frequently” (top); perceived benefit, on a 4-point scale ranging from ‘Would not improve’ to ‘Would greatly improve’ (center); and willingness to adopt, on a 5-point Likert scale from “strongly agree” to “strongly disagree” (bottom) is shown.

Clinician familiarity, perceived benefit, and willingness to adopt POC HPV testing, patient self-sampling, and rapid HPV self-testing with self-collected samples were quantitatively assessed in the survey (Fig 2).

Qualitative interviews provided insights on the specific benefits and limitations of these innovative screening modalities, reasons why clinicians are willing or unwilling to adopt them, and additional considerations for implementation of rapid HPV testing for cervical cancer screening (Table 2).

**Table 2:** Perceived benefits and limitations of rapid testing modalities for cervical cancer screening.

Clinician-led POC HPV testing	Patient HPV self-sampling	Patient HPV self-testing
**Benefits:** 1. Increase screening opportunities 2. Decrease patient costs 3. Decrease patient time spent at clinics 4. Ability to give immediate results, counsel and educate patients in real-time 5. Ensure follow up in same visit	1. Privacy and comfort of patients 2. Reduce key barriers to screening	1. Increase access to testing and cervical cancer screening 2. Potential way to encourage establishing relationship with provider
**Limitations:** 1. User errors and rapid test accuracy 2. Does not replace need for cytology 3. If done in the absence of a Pap test: Potential to miss non-HPV abnormalities	1. Unsure of patient ability to acquire adequate sample 2. Missing other abnormalities caught by Pap or pelvic exam 3. Patients may not return samples	1. Failure to report results to provider 2. Unsure of patient ability to correctly interpret results on their own 3. Potential for patients to neglect other preventive care

### Clinician POC HPV testing

Of all survey respondents, 49% (n = 110) frequently and 14% (n = 31) occasionally used POC tests for any purpose, 5% (n = 12) had used them in the past, 20% (n = 44) were familiar but had not used them, and 12% (n = 28) first heard about them in the survey (Fig 2A). Those who reported using POC testing mentioned using them for hemoglobin A1-C, cholesterols, strep, flu, urinalysis, pregnancy, HIV, and COVID testing. Most respondents (n = 201, 89%) believed POC HPV testing would improve cervical cancer screening coverage and/or follow-up for their patients either greatly or somewhat; (n = 17, 8%) believed it would not improve screening, and (n = 7, 3%) were unsure (Fig 2B). Most respondents (n = 186, 82%) strongly agreed or agreed that they “would support adopting POC HPV testing in [their] clinic[s]” assuming FDA approval. Among the remaining respondents, 16% (n = 35) were uncertain, and less than 2% disagreed (Fig 2C).

In qualitative interviews, participants shared that an HPV POC test could increase screening opportunities for their patients, *“The benefit would be to increase screening opportunities for women and be able to provide them with the information in real time about what their risks are cervical cancer and ensuring adequate follow up (Physician*, *Family Medicine*, *Female)*”. The ability to acquire results in real-time would reduce the need for laboratory-based testing potentially decreasing patient costs, as well as more accurately identifying which patients actually need additional screening, *“…probably decrease costs to patients*, *I would assume it might even decrease the time that they had to be in the clinic and it would probably increase the accuracy of this screening we actually needed to do*.*(Nurse Practitioner*, *Family Medicine*, *Female)”* Some clinicians also expressed that obtaining the testing information rapidly allows clinicians to provide more education about HPV and counsel patients on the results in real time, *“you can sit down and counsel them right immediately*. *(Nurse Practitioner*, *Obstetrician/Gynecologist*, *Female)”* Other reported benefits include: guaranteeing appropriate follow up care which includes testing, management, and implications of HPV, *“rapid information would give you another opportunity to educate patients and talk about follow up testing if needed*, *follow up management*, *and kind of the course and implications of HPV*. *(Physician*, *Obstetrician/Gynecologist*, *Female)”*.

Among reported concerns, clinicians felt that POC HPV testing may be limited by the need for cytology, *“They may just need a pap smear anyway*, *because we need to know what the cytology is*. *(Nurse Practitioner*, *Family Medicine*, *Female)”* Another perceived limitation was the potential to miss other non-HPV issues in the absence of a pelvic exam, *“There’s the potential that you would miss some type of abnormality or risk of cervical cancer not related to HPV*. *(Physician*, *Family Medicine*, *Female)”* Additionally, clinicians expressed concerns about user errors when conducting rapid tests, *“The negative test I would always be a little questionable about because you just got to make sure it’s done right and I’m not even saying that practitioners that do it are necessarily doing them right so there’s always that user error that kind of worries me a little bit*.*(Nurse practitioner*, *Family Medicine*, *Female)”*.

### Patient self-sampling

Among respondents, 29% (n = 65) reported frequently or occasionally using self-sampling with their patients for any condition, 7% used it in the past (n = 15), 38% (n = 86) were familiar with self-sampling but had not used it, and 26% (n = 59) first heard about it in the survey (Fig 2B). Among the clinicians that used self-sampling, they did so for the following tests: fecal immunochemical test (FIT) for colon cancer, sexually transmitted diseases, and bacterial vaginosis. Most (72%, n = 161) believed self-sampling would greatly or somewhat improve cervical cancer screening coverage and/or follow-up, while 15% (n = 34) did not believe it would improve screening and 13% (n = 30) were unsure. Half (50%) of respondents (n = 112) strongly agreed or agreed with adoption of patient self-sampling in their clinic, 35% (n = 78) were uncertain, 10% (n = 23) disagreed with adoption, and 5% (n = 12) strongly disagreed (Fig 1C).

Interviewees discussed perceived benefits of patient self-sampling, including being a less intimidating method that increases patient comfort, *“I think that many women*, *maybe most*, *would be relieved to be able to do a self-sample instead of having to have someone else take one (Physician*, *Family Medicine*, *Female)”*, especially for patients that may have had negative experiences and are more vulnerable as explained by one participant: *“So that’s something they could do at home or even in the clinic in private*, *they would like that*. *You know we have a lot of patients that have history of trauma and abuse*. *So*, *it’s nerve wracking to come in and be in such a vulnerable position*. *Yeah so I think self-sampling would be very helpful for that reason*.*(Nurse Practitioner*,*Obstetrician/Gynecologist*, *Female)”* Most clinicians felt that the privacy afforded by self-sampling would encourage cervical cancer testing in some patients, *“I think if the patient knew what she was doing*, *the preference would be to obtain the sample in the privacy of your own environment*, *and you pick where that is*.*(Physician*, *Obstetrician/Gynecologist*, *Male)”* Clinicians also reported that self-sampling could be a promising alternative for patients facing barriers that prevent them from going to a clinic, *“It’s clearly better than nothing*. *And I think for patients especially where the barrier is coming into clinic or getting time off work or finding childcare or whatever… (Physician*, *Obstetrician/Gynecologist*, *Female)”*.

Perceived limitations of self-sampling centered on patients’ ability to acquire an accurate sample, *“My concern is like*, *Where exactly are you sampling*? *You know*, *are they doing it correctly*?… *I guess*, *if you do education around how exactly to collect it or how to self swab*… *So just ensuring the patient does it correctly*, *basically*.*(Physician*, *Family Medicine*, *unspecified gender)”* Clinicians also expressed some concerns with at-home self-sampling because of the possibility of not being able to do a physical exam and missing other signs such as lumps, warts, or other abnormalities, *“I think we do a self-sampling at home*, *we missed that opportunity to do that visual and our physical exam on a patient to do their breast exam and maybe a lump that I may miss*. *So I think it may limit some of the other assessments and things that need to take place with that well*, *women’s exam*.*(Nurse Practitioner*, *Family Medicine*, *Female)”* as well as having limited provider- patient interaction in which the provider gives a complete array of care, *“I think the most ideal situation would be the patient’s in the office and you can kind of verbally go through it and in the room with them face to face*. *Because that way*, *I think you’re still ensuring that you’re getting like full comprehensive care*. *So not just the cervical cancer screening*, *but also that they’re getting all their other care needs addressed (Physician*, *Family Medicine*, *Female)”*. Clinicians also expressed concerns over not receiving the sample collected back for analysis, *“And so I don’t know if it’s an issue with the patient’s not completing the test or an issue with them*, *not mailing it in… I would have concerns about doing the test that way*. *(Physician*, *Family Medicine*, *Female)”*.

### Patient HPV self-testing with self-collected samples

Patient self-testing for any condition (mostly COVID) was reported with use frequently or occasionally by 30% (n = 68) of respondents, 8% (n = 19) only used it in the past, 32% (n = 73) were familiar but had not used it, and 29% (n = 65) first heard about it in this survey. Most respondents (70%, n = 158) believed patient self-testing would greatly or somewhat improve cervical cancer screening screening, while 14% (n = 31) did not believe it would improve screening and 16% (n = 36) were unsure. Regarding supporting adoption of patient self-testing for HPV “without needing to come into the clinic if the results are normal,” 48% were willing to adopt this method; 39% (n = 88) were uncertain; 7% (n = 15) and 6% disagreed or strongly disagreed with adoption (Fig 2). Of those respondents who were willing to adopt patient self-sampling, most (74%, n = 83) were also willing to adopt patient self-testing with self-collected samples.

In-depth interviews revealed perceived benefits of patient self-testing for HPV with self-collected samples for cervical cancer screening. Clinicians felt an important benefit of this method would be its potential to increase access to testing for those who are unwilling or unable to access a clinic for cervical cancer screening, and potentially encourage patients to establish contact with providers if they were to test positive on an HPV self-test: *“I think it would increase access to testing and a percentage of those people that get a positive test are not going to do anything about it*, *but a percentage of them are going to then establish care with a gynecologist or a physician*, *a primary care doctor and determine what next steps they need to take*. *So*, *there’s where the value of the self-run*, *self-interpreted test*, *could be*. *(Physician*, *Internal Medicine*, *Female)”* A self-test, as opposed to self-sampling alone, would also address clinician concerns over patients not mailing back a sample for analysis.

However, clinicians also identified limitations of this method such that patients could run the test at home but fail to report the results to the provider, *“If I’m talking to a patient in the clinic and I take ownership of ordering that test*, *and the patient goes home and runs it and doesn’t tell me what the result is*, *and I don’t have a clear system for following up on the result*, *when they show up in three years with cervical cancer that’s on me because I told them to do this test (Physician*, *Internal Medicine*, *Female)*.” Clinicians also repeatedly voiced concerns that patients screening at home may not be able to medically interpret the results, *“My concern would be that I have a 25 year old who would do a test and would be positive and she freaks out and ‘he gave this to me how did they give it to me*. *What does this mean’ and it’s going to create a lot of hassle (Physician*, *Internal Medicine*, *Female)*.*”* Furthermore, clinicians feared that patients would choose not to get in contact with a provider for follow-up or would forego preventive medical visits, *“So I worry that when you have someone screening at home*, *and running the test themselves*. *You’re missing that interpretation piece that makes sure the patient gets the follow up they need*. *(Physician*, *Internal Medicine*, *Female)”* … *“I think we just have to be careful that patients don’t think that self testing [for] HPV at home is going to supplant the need for preventive visits to their family physician or internist or gynecologist*. *(Physician*, *Family Medicine*, *Female)”*.

#### Considerations for implementation of rapid testing for cervical cancer screening

Through qualitative interviews, clinicians provided key considerations for the implementation of rapid testing (POC and self-testing) for cervical cancer screening. Clinicians expressed that they need more data on rapid testing for cervical cancer screening to gain a deeper understanding of the usefulness and the specific guidelines that come with the test, *“I’m curious to see*, *you know exactly how reliable it is … I would like to see more data behind it to see how useful it actually is*. *And you know I want to see exactly what the guidelines say about how frequently to screen and what to do with those results but as long as that’s all clear to me*. *I think that it [POC testing] would benefit my patients*. *(Physician*, *Family Medicine*, *Female)”* Regarding at-home self-testing, clinicians emphasized that patients must have the appropriate education on how to collect a reliable sample, *“the fundamental is does the patient know how to acquire a reliable specimen*. *(Physician*, *Obstetrician/Gynecologist*, *Male)”* Clinicians consistently mentioned that the self-test should come with instructions such as a *“home instruction video*” on how to collect the sample, as well as it being a simple tool for patients to use *“but it really needs to be very*, *very simple and very*, *very foolproof*. *Like there has to be some system or some apparatus or piece of plastic that lines things up*, *right so it goes in the right spot*. (Physician, Family Medicine, Male)” Clinicians mentioned that they preferred to report and explain the results to patients themselves which COVID showed could be implemented through telehealth visits, *“if this could be kind of like the patient would do the test at home*, *get the result*, *and then be on like a telehealth visit then they could get the results explained to them by telehealth… I’m all for telehealth visits like when*, *when people have to come to the office for like a birth control renewal after a year*. *It’s such a pain*, *and they’re so put out*, *and frustrated about it*. *(Nurse Practitioner*,*Obstetrician/Gynecologist*, *Female)”*.

## Discussion

This study had two objectives: 1) to examine whether COVID influenced clinician perspectives of rapid testing as a screening modality and 2) to assess clinician awareness, perceived benefits and limitations, and willingness to adopt POC HPV testing, patient self-sampling, and rapid HPV self-testing with self-collected samples for cervical cancer screening. Approximately half of the surveyed clinicians felt that experiences during the COVID pandemic had influenced their views on rapid testing as a screening modality. Of these, the majority were positively influenced, reporting greater public acceptability of rapid testing and positive impacts on patient care such as the ability to decrease barriers to screening; however, several reported strong concerns around the accuracy of rapid tests. Clinicians shared important considerations and lessons learned during COVID for the implementation of rapid testing for cervical cancer screening. According to the interviewees, these are needed to properly educate patients on how to collect a reliable sample and very clear, simple instructions for running a rapid test taking into consideration that some patients may be subject to social and health inequalities that are caused by low education, low technology access and low health literacy that could alter their ability to perform and interpret tests. Furthermore, the need for adequate communication with patients, both to explain test results and to initiate follow-up, was an important concern that some felt could be addressed through telehealth.

The vast majority (82%) of surveyed Indiana clinicians reported high acceptability and willingness to adopt rapid HPV testing at the POC, citing key benefits such as the ability to explain results to patients and initiate any necessary follow-up procedures in a single clinic visit; 89% felt rapid testing would improve cervical cancer screening. However, when it came to patient self-testing with self-collected vaginal swab samples, enthusiasm among clinicians diminished. While 70% felt self-testing would improve cervical cancer screening coverage and access, only 48% were willing to adopt this screening method, citing key concerns about patients’ ability to acquire a reliable sample and run the rapid test properly, and whether patients might be less likely to see their doctors for other preventive care.

Our findings align with a previous study of 350 American primary care providers in which 43% had heard of POC and 97% expressed interest in using POC cancer screening technologies after being presented with more information [26]. In a 2020 study of U.S. health experts, 44.6% of respondents had a positive experience with POC testing during the COVID pandemic and indicated that POC testing, in general and regardless of condition, was most beneficial for its improvement of patient management and most concerning for risk of overtesting [32]. Other studies of at-home screening interventions reported clinician concerns about whether patients would continue preventive care visits and receive follow-up care [4]. Our study identified similar concerns among Indiana clinicians, in addition to clinician uncertainty about the accuracy of rapid tests and patient self-sampling.

Understanding clinician familiarity and perspectives on rapid testing and self-sampling, and how these may have shifted with the increase in adoption of COVID rapid tests, is essential to address key concerns about the utility of HPV POC and self-testing as potential screening modalities, and to mitigate barriers to clinical adoption for cervical cancer screening. Clinician concerns about a patient’s ability to adequately self-sample could be addressed through increased dissemination and provider communication on existing evidence of the efficacy of patient-collected versus clinician collected samples [16–18]. The ‘Last Mile’ Initiative, developed by the National Cancer Institute is a public-private partnership between federal agencies, industry practitioners, and professional guideline organizations to validate HPV self-sampling testing and facilitate FDA approval and uptake in the US [33]. These efforts, in addition to the faster development and evaluation of rapid tests during COVID could provide a blueprint for the innovation of cervical cancer screening tools. Additionally, this key concern among clinicians informs the need for rapid HPV tests to include sample adequacy controls that can confirm the reliability of self-collected samples.

Regarding clinician concerns about the accuracy of rapid tests, an important consideration is the need to be clear about the purpose and goal of a rapid test. SARS-CoV-2 rapid tests, despite having lower sensitivity compared to PCR, accurately detect antigen levels during a person’s infectious period, and thus are invaluable tools for determining isolation periods to limit transmission [34]. For cervical cancer, a rapid test may never be as sensitive as PCR, but could detect high enough levels of HPV that correlate with higher cervical cancer risk [35], so as to avoid overdiagnosis of patients [36]. Thus, the purpose of these tests should be to triage patients so that we can identify those at highest risk who need immediate follow-up tests like cytology or colposcopy. Properly educating both providers and patients on the purpose of a rapid HPV test and the correct interpretation of results will be key for cervical cancer.

The widespread adoption of COVID rapid testing both at the POC and at-home with patient-collected samples, and increase in consumer preferences for these tools presents a key opportunity to implement alternative cervical cancer screening modalities that have the potential to address barriers among under-screened populations and reverse the trend of declining screening rates in the U.S. Such screening modalities can increase convenience for patients and address key sociocultural barriers among medically-underserved populations that may limit them from getting screened. The buy-in and willingness of healthcare providers to adopt these tools is necessary in order to take advantage of this window of opportunity to improve cancer screening. While this study focused on Indiana providers and may not be representative of other contexts across the US, our findings point to key opportunities to introduce cervical cancer screening innovations such as rapid HPV testing at the POC, and key concerns among clinicians that must be addressed before additional modalities like at-home testing could be implemented. A limitation of this study is that it focuses only on provider perspectives and lacks the patient perspective. The perspectives and preferences of patients for rapid HPV testing are essential to successful implementation and is the focus of future work. Furthermore, while many interviewed clinicians spoke of segments of their patient populations who were underserved, we did not specifically select clinicians who focus only on underserved populations nor did we systematically collect data about the interviewed clinicians’ patient populations. Clinicians who serve primarily underserved populations may have different opinions on HPV self-sampling and rapid testing modalities, which will be explored in future work. Leveraging the momentum of COVID-era healthcare innovation, regarding both the tools we use to screen and the ways to deliver these tools, will be key to addressing persistent and widening cervical cancer disparities in the US.
